# Case Report: Pediatric Philadelphia chromosome-positive T-lymphoblastic leukemia relapsing as chronic-phase CML after treatment discontinuation

**DOI:** 10.3389/fonc.2026.1874606

**Published:** 2026-07-07

**Authors:** Rongrong Dong, Xinying Dai, Yuan Feng

**Affiliations:** 1 Department of Laboratory Medicine, Qingdao Women and Children’s Hospital, Qingdao University, Qingdao, Shandong, China; 2 Department of Hematology, People’s Hospital of Qianxinan Prefecture, Xingyi, Guizhou, China; 3 Department of Laboratory Medicine, People’s Hospital of Qianxinan Prefecture, Xingyi, Guizhou, China

**Keywords:** *BCR::ABL1*, blast phase, chronic myeloid leukemia, Philadelphia chromosome, T-lymphoblastic leukemia, treatment adherence, tyrosine kinase inhibitor

## Abstract

**Background:**

Philadelphia chromosome (Ph)-positive T-lymphoblastic leukemia (T-ALL) is exceptionally rare. Distinguishing *de novo* Ph-positive T-ALL from T-lymphoid blast-phase chronic myeloid leukemia (BP-CML) can be highly challenging, yet this distinction has important therapeutic and prognostic implications. We report a pediatric patient initially diagnosed with *de novo* Ph-positive T-ALL who subsequently developed CML following treatment discontinuation.

**Case presentation:**

A 14-year-old boy presented with fever, leukocytosis, and generalized lymphadenopathy. Bone marrow examination revealed 81% blasts, and flow cytometric immunophenotyping confirmed T-ALL (cytoplasmic CD3^+^ CD5^+^ CD7^++^). Conventional cytogenetic analysis revealed a karyotype of 49,XY,−7,+8,t(9;22)(q34;q11.2),+15,+19,+mar[3], and real-time quantitative reverse-transcription polymerase chain reaction detected a b3a2 *BCR::ABL1* fusion transcript. A diagnosis of *de novo* Ph-positive T-ALL was rendered, and the patient achieved complete remission following induction chemotherapy with VICP (vincristine, idarubicin, cyclophosphamide, prednisone) plus imatinib. The patient declined both allogeneic hematopoietic stem cell transplantation and escalation to a second-generation tyrosine kinase inhibitor (TKI). After self-discontinuing imatinib without medical advice for approximately 18 months, he developed CML.

**Conclusions:**

This case highlights the diagnostic challenge of distinguishing *de novo* Ph-positive T-ALL from T-lymphoid BP-CML at initial presentation, particularly in pediatric patients. Accurate classification is essential because it has important implications for therapeutic decision-making and long-term management in the TKI era. The subsequent development of CML following TKI discontinuation strongly supports that the initial presentation represented lymphoid BP-CML rather than *de novo* Ph-positive T-ALL. This observation further underscores the importance of sustained TKI therapy and adherence in preventing disease recurrence and optimizing long-term clinical outcomes.

## Introduction

The Philadelphia chromosome (Ph) arises from a reciprocal translocation involving the *BCR* gene on chromosome 22 and the *ABL1* proto-oncogene on chromosome 9, leading to the formation of the *BCR::ABL1* fusion gene (1). This fusion transcript encodes a constitutively active tyrosine kinase with potent leukemogenic activity. The Ph chromosome is present in more than 95% of patients with chronic myeloid leukemia (CML) and in approximately 20-40% of patients with B-lymphoblastic leukemia (B-ALL), whereas its occurrence in T-lymphoblastic leukemia (T-ALL) is exceedingly rare (2–4). Ph-positive ALL typically follows an aggressive clinical course and has historically been associated with an unfavorable prognosis, characterized by short remission durations and inferior survival despite intensive chemotherapy (5).

Among cases of Ph-positive ALL, some represent CML presenting in lymphoid blast phase (BP-CML), whereas others arise as *de novo* acute leukemia. Distinguishing between these two entities can be challenging, particularly in pediatric patients. Both *de novo* Ph-positive T-ALL and T-lymphoid BP-CML are exceedingly rare, with only isolated case reports documented in the literature (6, 7). Here, we report a pediatric patient initially diagnosed with *de novo* Ph-positive T-ALL. Following treatment with chemotherapy and imatinib, the patient achieved complete remission but declined both bone marrow transplantation and a switch to a second-generation tyrosine kinase inhibitor (TKI). Approximately 18 months after discontinuing TKI therapy without medical supervision, the patient developed typical CML, providing compelling evidence that the initial presentation represented T-lymphoid BP-CML rather than *de novo* Ph-positive T-ALL.

## Case presentation

**Figure 1:**
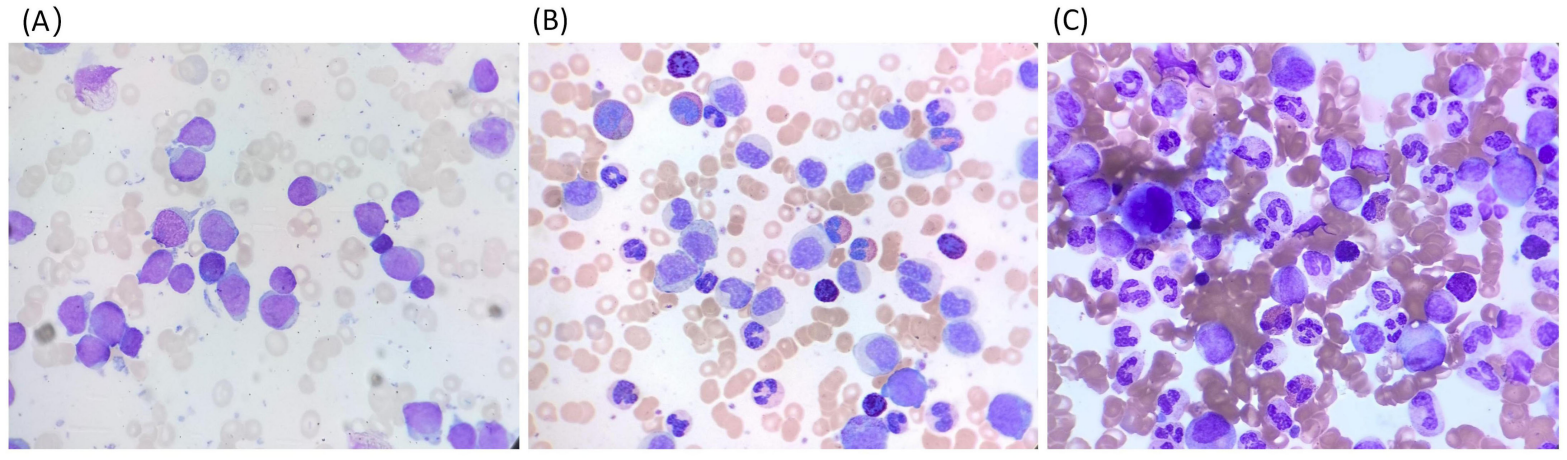
Bone marrow aspirate smear at the initial diagnosis showed numerous blasts **(A)**. Peripheral blood smear at relapse demonstrates frequent immature granulocytes, eosinophils, and basophils **(B)**. Bone marrow aspirate smear at relapse showed myeloid predominance with increased basophils and eosinophils, dwarf megakaryocytes, and no increased blasts **(C)**.

**Figure 2:**
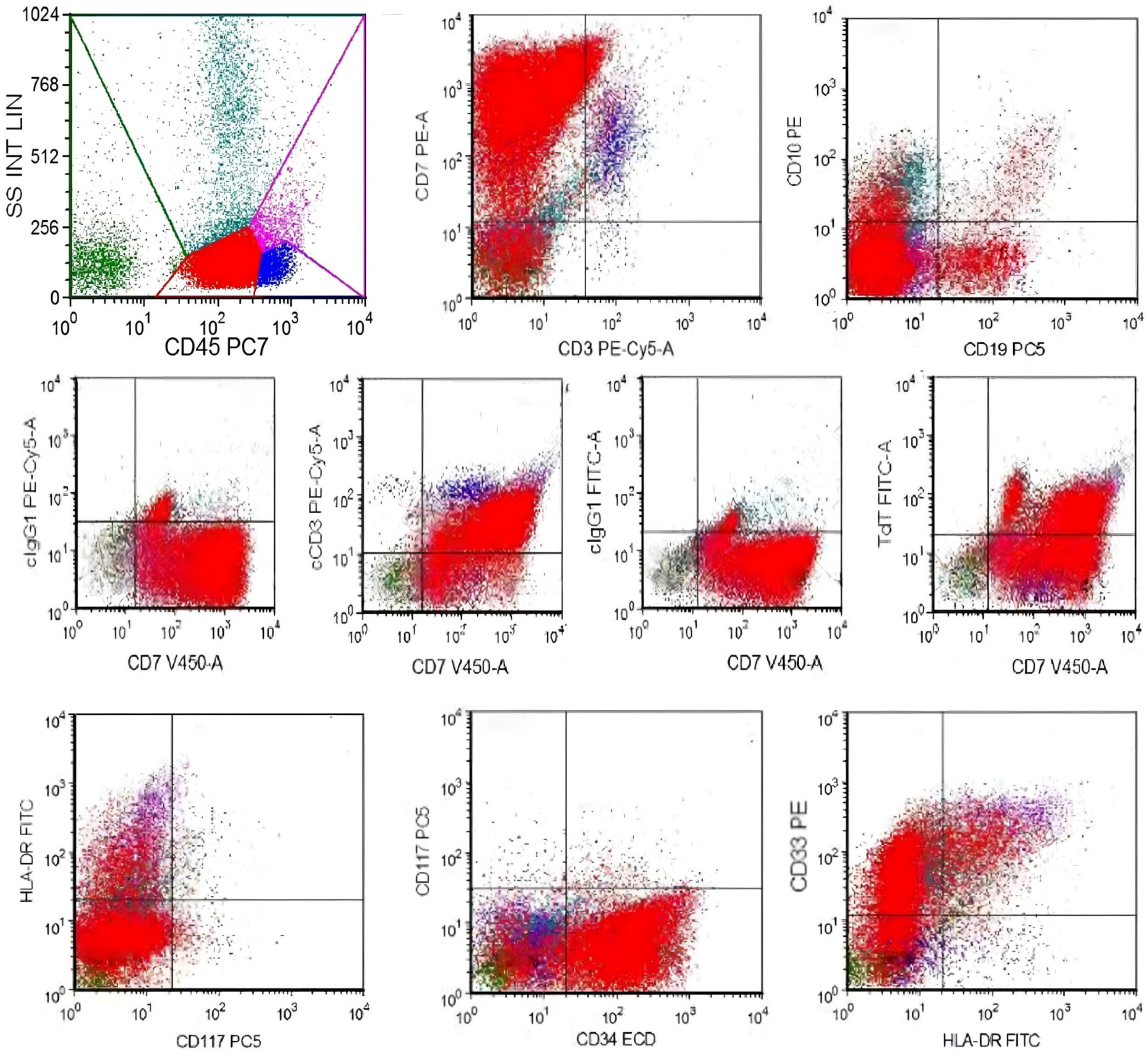
Results of flow cytometric analysis at initial diagnosis of T-lymphoblastic leukemia.

**Figure 3:**
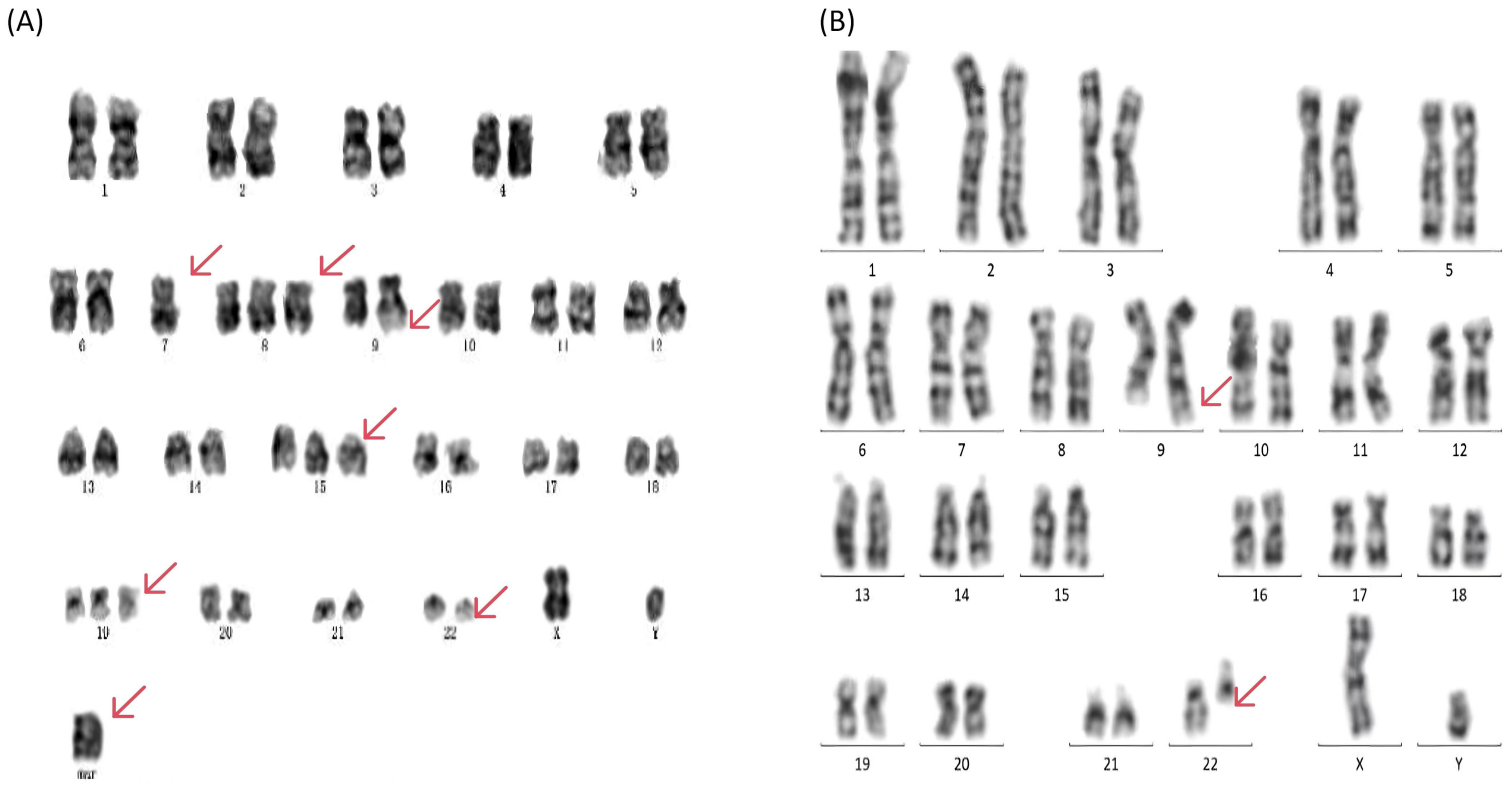
Karyotypes at initial diagnosis of T-lymphoblastic leukemia **(A)** and at relapse as CML **(B)**. Abnormal chromosomes are marked with red arrows.

In March 2019, a 14-year-old boy presented with fever and generalized lymphadenopathy. Physical examination revealed multiple enlarged lymph nodes involving the anterior and posterior cervical, submental, submandibular, retroauricular, occipital, supraclavicular, axillary, and inguinal regions bilaterally. Some lymph nodes were matted, firm, and mildly tender. Complete blood count (CBC) showed severe anemia with a hemoglobin (Hgb) level of 5.6 g/dL, a platelet (PLT) count of 162 × 10^9^/L, and leukocytosis with a white blood cell count (WBC) of 53.55 × 10^9^/L. The peripheral blood differential count demonstrated 70% blasts, 10% segmented neutrophils, 3% monocytes, 1% eosinophils, 1% basophils, and 15% lymphocytes. Bone marrow (BM) biopsy showed a nearly 100% cellular marrow extensively replaced by sheets of blasts. BM aspirate differential showed 81% blasts (**Figure 1A**), which were negative for myeloperoxidase by cytochemistry stain. Flow cytometric immunophenotyping identified a large population of T-lymphoblasts comprising 74.7% of total events with following immunophenotype: partial CD34^+^, CD117^-^, partial CD33^+^, few HLA-DR^+^, partial CD5^+^, CD7^++^, partial CD2^+^, CD8^-^, CD4^-^, CD3^-^, CD56^-^, cCD3^+^, partial nTdT^+^, few CD19^+^, few cCD79a^+^, very few cCD22^+^, few CD10^+^, and CD20^-^ (**Figure 2**). Chromosomal banding analysis (CBA) revealed a complex karyotype: 49,XY,−7,+8,t(9,22)(q34;q11.2),+15,+19,+mar[3] (**Figure 3A**). Real-time quantitative reverse-transcription polymerase chain reaction (RT-qPCR) detected a b3a2 *BCR::ABL1* fusion transcript. In the absence of characteristic morphologic features of CML, a diagnosis of *de novo* Ph-positive T-ALL was rendered.

The patient received induction chemotherapy with a VICP (vindesine, idarubicin, cyclophosphamide, prednisone) combined with imatinib and achieved complete remission as assessed by BM morphology and flow cytometric immunophenotyping. He subsequently received two cycles of consolidation chemotherapy with the CAM regimens (cyclophosphamide, cytarabine, 6-mercaptopurine) followed by one cycle of high-dose methotrexate. Allogeneic hematopoietic stem cell transplantation (allo-HSCT) was recommended but declined. The patient then continued maintenance chemotherapy and intrathecal prophylaxis, with serial cerebrospinal fluid flow cytometric analyses remaining negative for disease involvement.

In September 2020, the b3a2 *BCR::ABL1* became undetectable by RT-qPCR. Allo-HSCT was again recommended but was declined. Three months later, RT-qPCR testing showed reemergence of the b3a2 *BCR::ABL1* at a level of 0.02%. By December 2021, the *BCR::ABL1* transcript level had increased to 9.89%; however, BM morphologic examination and flow cytometric immunophenotyping showed no evidence of residual or recurrent T-ALL. A switch to a second-generation TKI was recommended but declined. The patient continued imatinib at a dose of 400 mg daily outside the hospital but subsequently discontinued imatinib treatment on his own and remained off therapy without regular follow−up.

In January 2024, approximately 18 months after self-discontinuing imatinib, the patient returned to our hospital with priapism and severe abdominal pain. CBC showed a Hgb level of 11.2 g/dL, a PLT count of 234 × 10^9^/L, and a WBC of 339 × 10^9^/L. The differential count revealed 11% blasts, 27% neutrophilic myelocytes, 8% neutrophilic metamyelocytes, 45% segmented neutrophils, 1% eosinophils, 6% basophils, and 2% lymphocytes (**Figure 1B**). BM biopsy showed a 100% cellular marrow with marked granulocytic hyperplasia, dwarf megakaryocytes, and grade 1 myelofibrosis. The BM aspirate differential count revealed a myeloid-to-erythroid ratio of 22.5:1 with elevated eosinophils and basophils (**Figure 1C**). Flow cytometric analysis revealed no residual or recurrent T-ALL, with only 0.87% myeloid blasts identified. CBA revealed a karyotype of 46,XY,t(9;22)(q34.1;q11.2)[20] (**Figure 3B**). RT-qPCR showed a b3a2 *BCR::ABL1* transcript level of 67.66%. No mutations were detected in the *ABL1* kinase domain. Based on these findings, a diagnosis of CML was established. The patient received cytoreductive therapy with hydroxyurea and cytarabine, underwent four leukapheresis sessions, and received surgical bloodletting with irrigation of the corpora cavernosa for management of priapism. One week later, the WBC count decreased to 51 × 10^9^/L. However, the patient declined further treatment and insisted on discharge. After discharge, the patient self-administered traditional herb medicine, the composition of which remained unknown. Unfortunately, he subsequently lost to follow-up once again.

In March 2025, approximately 13 months after CML relapse, the patient was readmitted to our hospital again because of a large, self-palpable left lower abdominal mass and intractable vomiting. Physical examination revealed palpable lymphadenopathy involving the posterior cervical, submandibular, and inguinal regions, as well as splenomegaly, with the spleen palpable 5 cm below the left costal margin. CBC showed a Hgb level of 10.1 g/dL, a PLT count of 207 × 10^9^/L, and a WBC count of 423 × 10^9^/L. The differential count revealed 2% blasts, 1% promyelocyte, 15% neutrophilic myelocytes, 17% neutrophilic metamyelocytes, 20% band neutrophil, 34% segmented neutrophils, 4% eosinophils, 5% basophils, and 2% lymphocytes. BM biopsy demonstrated a 40-50% cellular marrow with relative granulocytic hyperplasia and dwarf megakaryocytes. BM aspirate differential count revealed a myeloid-to-erythroid ratio of 47.8:1 with elevated eosinophils and basophils. Flow cytometric analysis again revealed no evidence of T-ALL, with only 1.10% myeloblasts detected. CBA revealed a karyotype of 46,XY,t(9;22)(q34.1;q11.2)[20]. RT-qPCR showed a b3a2 *BCR::ABL1* level of 83.48%. Collectively, these findings confirmed the diagnosis of chronic-phase CML (CP-CML). The patient declined lymph node biopsy. He was treated with hydroxyurea and flumatinib, a second-generation TKI. Unfortunately, he was discharged against medical advice two weeks later and was subsequently lost to follow-up once again.

## Discussion

CML is a multilineage hematopoietic stem cell neoplasm that may occasionally present initially in lymphoid BP. In contrast, Ph-positive ALL is generally considered a lineage-restricted lymphoid malignancy. Studies by Secker-Walker and Craig (8) demonstrated significant differences in disease-free survival and therapeutic management between patients with multilineage involvement and those with lymphoid-restricted disease. Therefore, accurate distinction between these two entities is of considerable clinical importance (9).

However, differentiation can be challenging because of substantial overlaps in their morphologic, immunophenotypic, and genetic features. Clinical findings favoring lymphoid BP-CML include a prior history of CML, older age, a prolonged symptomatic course, marked splenomegaly, increased granulocyte precursors, eosinophilia, basophilia, and detection of the *BCR::ABL1* fusion gene in both blast and maturing myeloid cell populations. In contrast, younger age, male sex, and extensive bone marrow blast infiltration are more suggestive of *de novo* ALL (10–12). Some investigators have used fluorescence *in situ* hybridization (FISH) to demonstrate *BCR::ABL1* positivity in neutrophils as evidence of multilineage involvement, thereby supporting a diagnosis of lymphoid BP-CML (13). Furthermore, the subsequent emergence of CP-CML in a patient initially diagnosed with Ph-positive ALL provides strong retrospective evidence that the original presentation represented lymphoid BP-CML rather than *de novo* Ph-positive T-ALL.

Prior to the TKI era, reversion from Ph-positive ALL to CP-CML was not infrequent due to the lack of effective targeted therapies (14). In the TKI era, however, reversion to CP-CML with typical hematological features is exceedingly rare. Hu et al. (15) reported an extremely unusual case of Ph-positive B-ALL in which the patient experienced recurrent lymphoid and myeloid relapses following treatment discontinuation, accompanied by distinct cytogenetic and molecular alterations in the lymphoid (Ph-positive B-ALL) and myeloid (CP-CML) diseases. This observation suggests that some patients initially diagnosed with Ph-positive ALL may, in fact, harbor underlying CML and subsequently manifest typical CML after achieving ALL remission, particularly when therapy is discontinued or inadequate.

Our case closely resembles that reported by Hu et al, as the patient relapsed into typical CML 18 months after self-discontinuing TKI therapy. At initial diagnosis, chromosome analysis was limited by poor culture quality, yielding only three evaluable metaphases. All three metaphases harbored additional copies of t(9,22) together with other cytogenetic abnormalities, including monosomy 7 (**Figure 3A**). In contrast, at relapse, all 20 analyzed metaphases demonstrated only the t(9;22) translocation without additional abnormalities. Consistent with the model proposed by Hu et al., we hypothesize that the patient initially presented in lymphoid BP-CML, wherein acquisition of additional copies of t(9;22) and other chromosomal abnormalities, including monosomy 7, contributed to T-lymphoblastic transformation, whereas the underlying myeloid disease arose from leukemic stem cells harboring t(9;22) as the sole cytogenetic abnormality. Supporting this hypothesis, Gong et al. (16) identified monosomy 7 as one of the most common additional chromosomal abnormality associated with CML progression and lymphoblastic transformation. Intensive chemotherapy administered for ALL likely eradicated the lymphoid clone carrying these secondary cytogenetic abnormalities but failed to eliminate the underlying leukemic stem cells harboring t(9;22) alone. Consequently, long-term TKI therapy remains essential for disease control; when treatment is discontinued or resistance develops, residual leukemic stem cells may re-expand and re-establish clinically overt CML.

Although the apparent transition from T-ALL to CP-CML may suggest a reduction in disease severity, it carries substantial clinical risks and highlights the critical importance of treatment adherence. Following eradication of ALL clones by intensive chemotherapy, sustained TKI therapy is required to suppress residual CML stem cells. Treatment interruption or dose reduction may compromise disease control by removing the selective pressure exerted by TKIs, thereby allowing residual leukemic stem cells to survive and undergo clonal expansion, a major mechanism of disease recurrence (17). While the disease reverted from an acute leukemic presentation to CP-CML, the latter requires lifelong management. Moreover, interruption of therapy may forfeit an optimal therapeutic window and increase the risk of progression to accelerated phase or BP with either myeloid or lymphoid immunophenotype, ultimately resulting in an unfavorable prognosis.

This case represents a rare manifestation of CML in the TKI era and underscores the diagnostic challenge of distinguishing *de novo* Ph-positive T-ALL from lymphoid BP-CML. Accurate classification is critical because it directly affects therapeutic strategies, prognostic evaluation, and long-term disease management. Furthermore, strict adherence to TKI therapy and regular clinical follow-up are essential to minimize the risk of disease recurrence and progression.

## Data Availability

The original contributions presented in the study are included in the article/supplementary material. Further inquiries can be directed to the corresponding author.

## References

[1] Cortes J Lang F . Third-line therapy for chronic myeloid leukemia: current status and future directions. J Hematol Oncol. (2021) 14:44. doi: 10.1186/s13045-021-01055-9 33736651 PMC7976694

[2] Arber DA Orazi A Hasserjian R Thiele J Borowitz MJ Le Beau MM . The 2016 revision to the World Health Organization classification of myeloid neoplasms and acute leukemia. Blood. (2016) 127:2391–405. doi: 10.1182/blood-2016-03-643544 27069254

[3] Jabbour E Kantarjian H . Chronic myeloid leukemia: 2020 update on diagnosis, therapy and monitoring. Am J Hematol. (2020) 95:691–709. doi: 10.1002/ajh.25792 32239758

[4] Hunger SP Mullighan CG . Acute lymphoblastic leukemia in children. N Engl J Med. (2015) 373:1541–52. doi: 10.1056/NEJMra1400972 26465987

[5] Foà R Chiaretti S . Philadelphia chromosome-positive acute lymphoblastic leukemia. N Engl J Med. (2022) 386:2399–411. doi: 10.1056/NEJMra2113347 35731654

[6] Raanani P Trakhtenbrot L Rechavi G Rosenthal E Avigdor A Brok-Simoni F . Philadelphia-chromosome-positive T-lymphoblastic leukemia: acute leukemia or chronic myelogenous leukemia blastic crisis. Acta Haematol. (2005) 113:181–9. doi: 10.1159/000084448 15870488

[7] Gong Z Xie W Wang W Chen Z Xu J Yuan J . T-lymphoid or T/myeloid blast phase of chronic myeloid leukemia in the era of tyrosine kinase inhibitor therapy: a report of 14 cases. Int J Lab Hematol. (2017) 39:e45–50. doi: 10.1111/ijlh.12605 27863007

[8] Secker-Walker LM Craig JM . Prognostic implications of break-point and lineage heterogeneity in Philadelphia-positive acute lymphoblastic leukemia: a review. Leukemia. (1993) 7:147–51. 8426467

[9] Short NJ Kantarjian H . Using immunotherapy and novel trial designs to optimise front-line therapy in adult acute lymphoblastic leukaemia: breaking with the traditions of the past. Lancet Haematol. (2023) 10:e382–8. doi: 10.1016/s2352-3026(23)00064-9 37003279

[10] Shah S Kundu R Mishra R Mukherjee S Singh A . A rare case of Philadelphia-positive (P210BCR-ABL1) T-cell acute lymphoblastic leukemia/lymphoma associated with minimal residual disease persistence after intensive chemotherapeutic approaches. Leuk Res Rep. (2024) 21:100456. doi: 10.1016/j.lrr.2024.100456 38572397 PMC10987326

[11] Efstathopoulou M Zoi K Siakantaris MP Koumbi D Zannou A Triantafyllou EF . A case report of chronic myelogenous leukemia presenting as blastic crisis with a T-cell acute lymphoblastic leukemia phenotype: awareness of a rare entity. Acta Haematol. (2023) 146:531–9. doi: 10.1159/000529911 37557081

[12] Jain P Kantarjian H Jabbour E Kanagal-Shamanna R Patel K Pierce S . Clinical characteristics of Philadelphia positive T-cell lymphoid leukemias-(De novo and blast phase CML). Am J Hematol. (2017) 92:E3–4. doi: 10.1002/ajh.24579 27727470 PMC5551407

[13] Kamoda Y Izumi K Iioka F Akasaka T Nakamura F Kishimori C . Philadelphia chromosome-positive acute lymphoblastic leukemia is separated into two subgroups associated with survival by BCR-ABL fluorescence in situ hybridization of segmented cell nuclei: report from a single institution. Acta Haematol. (2016) 136:157–66. doi: 10.1159/000445972 27537935

[14] Anastasi J Feng J Dickstein JI Le Beau MM Rubin CM Larson RA . Lineage involvement by BCR/ABL in Ph+ lymphoblastic leukemias: chronic myelogenous leukemia presenting in lymphoid blast vs Ph+ acute lymphoblastic leukemia. Leukemia. (1996) 10:795–802 8656674

[15] Hu S Jabbour EJ Hu CY Tang G Wang W Medeiros LJ . Recurrent lymphoid and myeloid relapses due to treatment cessations reveal natural history of Ph-positive B-ALL and pose a diagnostic challenge. Am J Hematol. (2024) 99:721–6. doi: 10.1002/ajh.27210 38240333

[16] Gong Z Medeiros LJ Cortes JE Chen Z Zheng L Li Y . Cytogenetics-based risk prediction of blastic transformation of chronic myeloid leukemia in the era of TKI therapy. Blood Adv. (2017) 1:2541–52. doi: 10.1182/bloodadvances.2017011858 29296906 PMC5728641

[17] Rousselot P Charbonnier A Cony-Makhoul P Agape P Nicolini FE Varet B . Loss of major molecular response as a trigger for restarting tyrosine kinase inhibitor therapy in patients with chronic-phase chronic myelogenous leukemia who have stopped imatinib after durable undetectable disease. J Clin Oncol. (2014) 32:424–30. doi: 10.1200/JCO.2012.48.5797 24323036

